# The Impact of the Ecosystem on Health Literacy Among Rural Communities in Protected Areas: Protocol for a Mixed Methods Study

**DOI:** 10.2196/51851

**Published:** 2024-01-29

**Authors:** Nor Aziah Abd Kadir, Amirah Azzeri, Hafiz Jaafar, Mohd Iqbal Mohd Noor, Zurina Kefeli

**Affiliations:** 1 Faculty of Medicine and Health Sciences Universiti Sains Islam Malaysia Negeri Sembilan Malaysia; 2 Faculty of Business and Management Universiti Teknologi MARA Cawangan Pahang, Kampus Raub Raub Malaysia; 3 Institute for Biodiversity and Sustainability Development Universiti Teknologi MARA Shah Alam Malaysia; 4 Faculty of Economics and Muamalat Universiti Sains Islam Malaysia Negeri Sembilan Malaysia

**Keywords:** ecosystem, health literacy, protected areas, Net-Map, quality of life, rural communities, protocol

## Abstract

**Background:**

Protected areas are crucial for the maintenance of human health and well-being. They aim to preserve biodiversity and natural resources to secure various ecosystem services that are beneficial to human health. Their ecological characteristics can influence local health literacy. Typically, communities surrounding protected areas have limited economic opportunities due to restriction policies to protect the ecosystem, resulting in socioeconomic disparities. The local community faces obstacles in gaining access to health care facilities and health information due to these limitations. It is difficult for them to locate, comprehend, and apply information and services to make better health-related decisions for themselves and others.

**Objective:**

This study protocol examines the impact of the ecosystem on health literacy among rural communities in protected areas.

**Methods:**

This study comprises 5 phases. In phase 1, we conduct a systematic review to identify the issue of health literacy in protected areas. In phase 2, we will collect data from stakeholders in a protected area of Pahang National Park and analyze the results using Net-Map analysis. In phase 3, we will conduct a survey among the adult community in Pahang National Park related to health literacy, socioeconomic status, health expenditure, and quality of life. In phase 4, informed by the results of the survey, we will determine suitable intervention programs to improve health literacy through a focus group discussion. Finally, in phase 5, we will conduct a costing analysis to analyze which intervention program is the most cost-effective.

**Results:**

This study was funded by Universiti Sains Islam Malaysia (USIM) and strategic research partnership grants, and enrollment is ongoing. The first results are expected to be submitted for publication in 2024.

**Conclusions:**

This is one of the first studies to explore health literacy among rural communities in protected areas and will provide the first insights into the overall level of health literacy in the protected community, potential determinants, and a suitable intervention program with expected cost analysis. The results can be used to promote health literacy in other protected areas and populations.

**Trial Registration:**

International Standard Randomized Controlled Trial Number Registry ISRCTN40626062; http://tinyurl.com/4kjxuwk5

**International Registered Report Identifier (IRRID):**

PRR1-10.2196/51851

## Introduction

Good health and well-being can be achieved with adequate health literacy [1,2]. Adequate health literacy indicates that the individual can obtain, process, understand, and apply basic health information and services needed to make appropriate health decisions [3]. Higher levels of health literacy allow people to make better health decisions [4], be more committed, and do their jobs more efficiently [5]. Patients must have enough information about their health to leverage it and make well-informed decisions regarding getting health treatments and medicine. In the context of preventive health care, an adequate level of health literacy helps ensure that patients get the most out of their health investments and make the best use of resources [6]. According to the World Health Organization (WHO), people residing in rural areas often experience socioeconomic inequity and lower educational attainment due to imperfect living conditions. These areas are cut off from urban centers and have limited facilities [7,8]. The varying ecosystems within the population may influence different levels of health literacy.

People living in the protected areas often have limited health literacy [9]. The government designates protected areas for conservation purposes. Thus, less emphasis is placed on new land development for farming, housing, and infrastructure [10-12]. As a result, populations encounter challenges in gaining better access to networks, health facilities, the internet, and other resources. This limitation, in turn, adversely affects their quality of life [9]. The health outcomes, living conditions, and education levels of the population residing in rural areas lag significantly behind those living near urban centers. This disparity places people in rural areas at a disadvantage when it comes to making informed health decisions, as they have limited knowledge in this regard.

Accordingly, policy makers are putting in place and advocating for health programs and awareness campaigns to improve the health literacy status among populations in rural and protected areas [13]. However, this strategy is hard to implement in communities that live in protected areas as they have limited educational backgrounds. They struggle to access, obtain, understand, and apply health information in their daily lives. Moreover, they are often unable to access health information when they are sick. Indigenous people often turn to traditional remedies, consult shamans, or opt for inaction with the hopes that the illness will recover on its own [14]. The limited knowledge of hygiene among indigenous people has prompted the government to develop a specialized syllabus for indigenous children at primary school. This curriculum focuses on teaching essential practices, such as washing hands, proper toilet usage, and dental hygiene. Understanding the impact of protected areas on health literacy in local communities is crucial to sustaining their way of life and health. However, the level of health literacy and its response to protected areas varies, depending on factors such as the type of protected area, how policies are planned, and how they are implemented [15].

Research on health literacy among people who live in protected areas is important for understanding their needs for access to health care, disease prevention, health promotion, and health care. It will also help elucidate how to meet these needs in the best and most effective way. Nevertheless, thus far, there has been limited research on health literacy and health issues among people in protected areas considering 4 perspectives (ie, that of government entities, knowledge institutions, civil agencies, and local communities). Accordingly, this paper proposes a study that will examine health literacy and health status of communities in protected areas. The study protocol can serve as a model for similar investigations in protected areas in other countries.

Based on the available evidence, this study aims to (1) conduct a systematic review to understand the health literacy status among rural communities surrounding protected areas; (2) explore stakeholders’ perceptions of health literacy status among rural communities surrounding protected areas; (3) determine the health literacy status and its associated factors among rural communities located near protected areas; (4) develop a new health literacy intervention program for rural communities near protected areas; and (5) conduct a cost structure analysis of the new health literacy intervention programs for rural communities surrounding protected areas.

## Methods

### Background and Conceptual Model

This study adopts a mixed methods approach to investigate health literacy.

**Figure 1:**
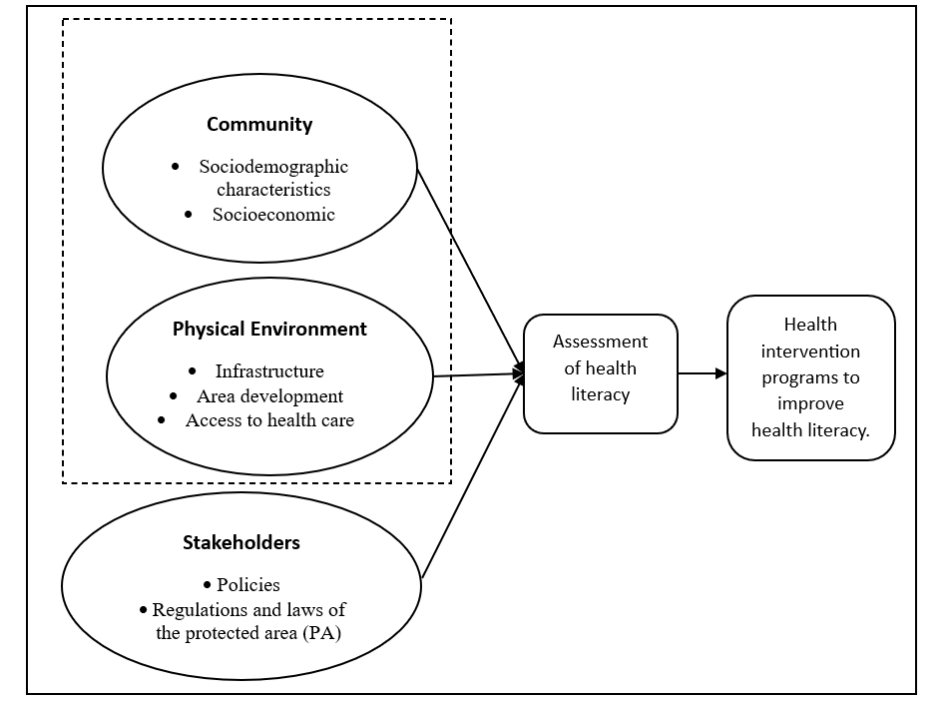
Conceptual model of the study.

Our understanding of health literacy is underpinned by the four core dimensions of the Health Literacy Model [16]: (1) accessing, (2) understanding, (3) appraising, and (4) applying health information. Based on this model, this study will explore the impact of the ecosystem on health literacy among rural communities in protected areas. Figure 1 explains the conceptual model for this study.

### Study Site

This study comprises one of Malaysia's protected areas, Pahang National Park (PNP). There are 53 national and state parks in Malaysia, with PNP being among the country’s most important and best-protected conservation areas. The region has diverse populations, including Malay and indigenous communities. As per the Jabatan Kemajuan Orang Asli (JAKOA; translated to Department of Orang Asli Development) [17], Pahang has the highest number of indigenous communities (37.9%) in Malaysia. Because the PNP site fulfills the criteria for this research (ie, the implementation of a protected area and the presence of legal or other formal mechanisms promoting such an area), we selected it as our study area.

### Research Design

This study contains 5 phases consisting of identifying the health literacy issues in this area, confirming the issues, testing the issues, and preventing and analyzing the issues. The 5 phases will be carried out based on the 5 objectives.

### Phase 1: Identifying Issues Through a Systematic Review

#### Overview

In phase 1, we will review the literature to understand the health literacy status among rural communities surrounding protected areas. This phase will involve conducting a systematic review following the PRISMA (Preferred Reporting Items for Systematic Reviews and Meta-Analyses) guidelines.

#### Approach

The systematic review will use a standard search string using the PICO (Population, Intervention, Comparison, and Outcome) structure. For the keyword search, we will use PubMed, Scopus, and Web of Science databases. Later, 2 reviewers will be appointed to appraise all selected studies. The result will compile the highest quality studies relevant to the study objectives.

#### Eligibility

The selection of results will be based on the following eligibility criteria: (1) full articles written in English and (2) articles published between 2012 and 2022. The exclusion criteria include (1) gray literature and (2) articles outside the scope of our research. The screening process will consider the qualifying requirements established.

#### Quality Assessment

First, 2 reviewers will conduct a quality assessment using a set of study quality assessments checklist. The checklist consists of eight evaluation criteria: (1) research goals, (2) study design, (3) study outcomes, (4) sample size calculations, (5) analysis of findings, (6) variance estimates for the primary results, (7) adequate reported results, and (8) conclusions. A quality score is one way to incorporate quality into the review process [18]. The reviewers will assign 2 points to articles that meet all criteria, 1 point if they fulfill some requirements, and 0 points if they do not. Subsequently, the quality score will be calculated by aggregating all elements from the quality assessment tool to provide an overall single score. In case of disagreements, a third reviewer will be appointed.

#### Data Coding and Analysis

This study employs a thematic synthesis, utilizing a 2-step coding procedure wherein reviewers will code each line of text from the articles separately for its meaning and substance. Before finishing this step, we will analyze every text associated with a particular code to ensure that the interpretation is consistent.

### Phase 2: Exploring Stakeholders’ Perceptions of Health Literacy Status in Rural Communities Surrounding Protected Areas

#### Overview

Phase 2 will use focus group discussions (FGDs) and in-depth interviews to achieve objective 2, which is to obtain stakeholders' opinions on health literacy issues among the community in PNP. The results will support the findings for objective 1.

#### Sample Selection

The selection of the stakeholders will be based on 3 categories, namely, government entities, knowledge-generating institutions, and civil society organizations [19,20]. However, the number of these actors will vary. This method will use purposive sampling to obtain a precise sample size. The expected actors can nominate any other actors with potential contributions to PNP's health literacy issue. This process will be halted when the answers given by the respondents are saturated [21].

#### Data Coding and Analysis

Data extraction and coding will be done for all Net-Map exercises, with a focus on thematic analysis utilizing information from the interviews. This analysis will mainly involve mapping activities and explaining the impact of protected areas on health literacy. The thematic analysis will follow a 2-step coding process. First, a line-by-line review of the interview data will be conducted to identify the key impacts related to the study objectives. Subsequently, each article will be assigned a code label to cluster all the information into a common theme.

### Phase 3: Identifying Health Literacy and Its Associated Factors in Rural Communities Surrounding Protected Areas

#### Participants and Sampling

This study will focus on the PNP. It covers 2477 square kilometers and was the country’s first national park established in Malaysia between 1938 and 1939, originally known as the King George V National Park. Following Malaysia's independence from British rule in 1957, the park was renamed. It is comprised of 3 protected areas in the states of Pahang, Kelantan, and Terengganu to form the central spine of Peninsular Malaysia. The national park has a reputation for being one of the world's oldest tropical rainforests, with an estimated age of 130 million years and a size of 4343 square kilometers.

Local communities in PNP are composed of Malay and Orang Asli (Batek and Semak Beri ethnicities). This location meets the criteria for this study because a protected area has been established and legal and other institutional measures have been established to promote them. The Pahang Department of Wildlife and National Parks (PERHILITAN) manages the PNP. The area is not open to the public except for those who have received prior approval from the authority for research purposes and minimal tourism activity at Kuala Tahan. Currently, the wildlife reserve contains a single research center that serves 2 distinct goals. The national park was originally established at Ulu Tembeling, with the first superintendent's office situated at Kuala Tahan. The southern section of PNP features a river boundary of over 80 kilometers along Sungai Tembeling, the Hulu Tembeling basin's principal river. Indigenous people inhabit the national park, while the Malay community resides near the river. For this study, respondents were drawn from the Malay and indigenous communities from the provinces of Hulu Tembeling and Tembeling Tengah. Table 1 shows the total number of respondents.

Respondents aged 18 years and above may vary in health status, including those with disabilities. A stratified random sampling approach (based on villages) will be employed for both the Hulu Tembeling and Tembeling Tengah provinces. All villages will be identified and visited. The choice of houses in the village will be selected randomly based on the number of houses given by the head of the village (Tok Empat and Tok Batin) and the inclusion criteria among the respondents, such as being able to read, having no cognitive disabilities, and being willing to participate in the survey.

**Table 1:** The total number of respondents and sample in Pahang National Park (PNP).

Name of village	Race	Province	Head of households (N=2525), n (%)	Sample (N=400), n (%)
Kg Pagi	Malay	Hulu Tembeling	95 (3.8)	15 (3.8)
Kg Kuala Sat	Malay	Hulu Tembeling	142 (5.6)	22 (5.6)
Kg Bantal	Malay	Hulu Tembeling	155 (6.1)	25 (6.1)
Kg Gusai	Malay	Hulu Tembeling	70 (2.8)	11 (2.8)
Kg Bukit Mat Daling	Malay	Hulu Tembeling	141 (5.6)	22 (5.6)
Kg Kuala Tahan	Malay	Tembeling Tengah	657 (26.0)	104 (26.0)
Kg Gol/Lik	Malay	Tembeling Tengah	40 (1.6)	6 (1.6)
Kg Merting/Lubok Payung	Malay	Tembeling Tengah	111 (4.4)	18 (4.4)
Kg Labu/Jong Berlabuh	Malay	Tembeling Tengah	163 (6.5)	26 (6.5)
Kg Pasir Sia/Air Hitam	Malay	Tembeling Tengah	86 (3.4)	14 (3.4)
Kg Selimbar/Chebong	Malay	Tembeling Tengah	58 (2.3)	9 (2.3)
Felda Sg Retang	Malay	Tembeling Tengah	509 (20.2)	81 (20.2)
Kuala Atok	Batek	Tembeling Tengah	35 (1.4)	6 (1.4)
Sungai Tiang	Semoq Beri	Tembeling Tengah	60 (2.4)	10 (2.4)
Sungai Tekal	Semoq Beri	Tembeling Tengah	83 (3.3)	13 (3.3)
Bukit Gam	Batek	Tembeling Tengah	22 (0.9)	3 (0.9)
Sg Keniam	Batek	Tembeling Tengah	35 (1.4)	6 (1.4)
Jeram Aur	Batek	Tembeling Tengah	13 (0.5)	2 (0.5)
Jeram Dedari	Batek	Tembeling Tengah	17 (0.7)	3 (0.7)
Sungai Yong	Batek	Tembeling Tengah	18 (0.7)	3 (0.7)
Sg Tabung/ Teresek	Batek	Tembeling Tengah	15 (0.6)	2 (0.6)

#### Sample Size

Two approaches will be considered to calculate the optimal sample size (ie, using the odds ratio [OR] and prevalence). The selection will be based on the method producing the higher sample size. For the OR, the sample size will be calculated using the OR of health literacy in Malaysia [22], identified as 3.41 with a 95% CI. We will use the Open Epi Software (version 3.01) to calculate the sample size. With an OR of 3.41, the estimated sample size is 310 as shown in Table 2. Furthermore, Table 3 shows the comparison of sample sizes for exposed and nonexposed groups in Kelsey and Fleiss methods.

In contrast, the second method uses prevalence. In Malaysia, the health literacy prevalence among individuals aged 18 years and above is 35.5% [23]. Using this percentage, the minimum sample size calculated for this study is 323 people for a 95% CI. Since the prevalence sample size is higher than the OR, we decided to use it as our sample size. Considering a dropout probability of approximately 10%, the total sample size needed for this study is about 355 people. However, we will distribute a sample size of 400 people based on the ratio calculated. We estimate that out of 400 adults, approximately 200 households will need to be interviewed based on the assumption that 1 household has 2 adults. To ensure that the provinces within the national park are well represented, the percentage of the selected population is determined to be 24%, 64%, and 12% for Malay in Hulu Tembeling, Tembeling Tengah, and indigenous people, respectively. Using the stratified random sampling techniques, the number of samples allocated for each village is outlined in Table 1.

**Table 2:** The estimated sample size.

Variables	Values, n
Sample size, n	310
Two-sided significance level (1-alpha)	95
Power (1-beta, % chance of detecting)	80
Ratio of sample size, unexposed/exposed	1
Percent of unexposed with outcome	5
Percent of exposed with outcome	15
Odds ratio	3.4
Risk/prevalence ratio	3
Risk/Prevalence difference	10

**Table 3:** Comparison of sample sizes for exposed and nonexposed groups in Kelsey and Fleiss methods.

Variables	Kelsey (n=274), n (%)	Fleiss (n=272), n (%)	Fleiss with CC^a^ (n=310), n (%)
Sample size, exposed	137 (50)	136 (50)	155 (50)
Sample size, nonexposed	137 (50)	136 (50)	155 (50)

^a^CC: continuity correction.

#### Inclusion and Exclusion Criteria

Participants in this study are required to be adults aged 18 or above and will be recruited after providing consent. Age verification will be based on their ID cards. Potential respondents may either be healthy or have various physical disabilities and demonstrate a willingness to participate. Additionally, they must be able to communicate in the Malay language. However, people with cognitive impairments will be excluded from the study.

#### Tools and Instruments

This study will comprise 3 evaluations: health literacy, health, and socioeconomic status. There are 8 parts to the questionnaire, which are outlined in Table 4.

**Table 4:** Components of the questionnaire.

Component	Description
Part 1: Housing and the environment (head of household only)	Part 1 will capture family structure, household transportation, and water supply.
Part 2: Household income (head of household only)	Part 2 will record the household's annual income from all sources.
Part 3: Sociodemographic characteristics (all household members, including the head of household)	Part 3 will capture sociodemographic information such as date of birth, ethnicity, education, employment status, relationship status, and so on.
Part 4: Health care expenditure and utilization (all household members, including the head of household)	Part 4 questions will capture data on health payments, health care service utilization, traditional and complementary medicine practice, and health care expenditure.
Part 5: Modifiable lifestyle factors (all household members, including the head of household)	Part 5 will ask about tobacco consumption, nutrition, physical activity, and medical conditions.
Part 6: Medical information (all household members, including the head of household)	Part 6 will measure the height, weight, waist circumference, and hip circumference. The calibrated vertical SECA portable 217 stadiometer (SECA GmBH & Co KG) will be used to measure respondents’ height, the calibrated SECA 813 digital electronic weighing scale will measure weight, and the SECA 201 ergonomic measuring tape will measure waist (abdominal) and hip circumferences.
Part 7: Quality of life (all household members, including the head of household)	Questions in part 7 will assess the participant's quality of life using standard and generic methods.
Part 8: Health literacy status	Health literacy status will be determined using the HLS-SF-Q12^a^ [24]. The perceived difficulty of each health-related task is rated on a 4-point Likert scale (1=*very difficult*, 2=*difficult*, 3=*easy*, and 4=*very easy*), with a possible lowest mean score of 1 and a possible highest mean score of 4. The questions consider the 4 competencies of an individual when dealing with health-relevant information (access/obtain, understand, appraise/judge/evaluate, and apply/use health information) to form a judgment and make health-related decisions for the 3 domains of health care, disease prevention, and health promotion.

^a^HLS-SF-Q12: 12-item Short-Form Health Literacy Questionnaire.

#### Reliability, Validity, and Trustworthiness

Reliability indicates whether the results can be consistently reproduced or not. In this study, internal consistency is evaluated through the Cronbach α value, with a threshold set at greater than or equal to .70 for satisfactory reliability. At the same time, validity will measure the validity and accuracy of the questionnaire. Face and content validity will be used to measure the validity of the questionnaire. Notably, this study incorporates established questionnaires with preestablished reliability and validity from different sources [22,25].

#### Data Collection Procedure

A total of 8 interviewers will be required to conduct the survey, working in pairs. For security and dialect reasons, a local representative will be appointed to accompany each team. Before the data collection, the ground staff will introduce the interviewers to the household members to ensure that the head of the household and other members are present and grant permission for the interviewers to enter their personal premises. Once approval is obtained, the interviewer will explain the nature of the study based on the participants’ information sheet, which outlines the key details. Subsequently, the information sheet will be provided to the participants for their reference. In addition, the interviewers will reassure the participants that the information provided in the study will remain confidential and their participation is entirely voluntary. The face-to-face interview will begin shortly after the head of the household and other household members have signed the consent form. During the interview, the respondents’ height and weight will be measured, contingent on suitability and participant preference. If the head of the household is absent during the visit, the house will be marked on the map, and a second visit will be arranged for the next day. However, the house will be excluded if the head of the household is still not present during the second visit.

#### Data Analysis Procedure

The data analysis procedure will involve both descriptive and analytical analyses. Initially, a descriptive analysis will focus on the sociodemographic characteristics of the study population. We will examine the distribution of study variables by calculating frequency and percentage, along with reporting mean and SD. Since the outcome of interest is categorical, nonparametric tests will be used. Following the approach outlined in [26], responses categorized as inadequate and problematic will be grouped as inadequate (0), while those categorized as sufficient and excellent will be classified as adequate (1) during the analysis.

For the analytical analysis, we will carry out both bivariate and multivariate analyses to explore the determinants of health literacy among both the study population residing whining the PNP and the population living outside the PNP. This comparative approach will aim to determine which ecosystem has adequate health literacy. The significance level has been set to *P*<.05. The analytical analysis will be conducted using SPSS software (version 28.0, IBM Corp).

### Phase 4: Developing a New Health Literacy Intervention Program for Rural Communities in the Protected Area

#### Overview

Phase 4 will be conducted via FGD discussion among the stakeholders.

#### Data Collection Procedure

The data collection process will be based on the opinions of the stakeholders and field experts. We will invite them to the seminar and sharing session to discuss the sustainability of the community near PNP. An invitation letter will be sent to the stakeholders and experts to confirm their willingness to participate in the discussion. They will include representatives from the Ministry of Health, JAKOA, Department of Wildlife and National Parks, heads of villages (Tok Empat and Tok Batin), nongovernmental agencies (NGOs), and academicians who are Taman Negara community health experts. During the seminar and sharing session, they will discuss mitigation measures that are suitable and effective in enhancing the health literacy status of the rural community.

#### Eligibility

The eligibility criteria for the intervention programs will be based on several factors, including the capacity of these programs, the financial support they received, their geographical location, prior experience in conducting health literacy programs, and willingness to participate. To account for differences in intervention activities, epidemiology, and target populations, we will limit our selection to only interventions that target communities located within the protected areas.

#### Data Analysis Procedure

During the discussion session, stakeholders will provide information regarding suitable intervention programs to improve health literacy at PNP using Logical Framework Analysis (LFA). The core components of LFA are outlined in Table 5.

**Table 5:** The core components of Logical Framework Analysis (LFA).

Component	Description
General objectives (goal)	This is the project's overarching goal. The project's benefits are laid forth in the objective goal for the beneficiaries.
Project purpose	The target group's plan of action to effect the desired change is expressed in the purpose. The project purpose frequently refers to modifying the target group's behavior because they use the services or goods the project offers.
Output	The project offers the products, services, and goods to the intended audience. The project is accountable for these outputs.
Activities	The project's processes for delivering the various goods, services, and products are outlined.

### Phase 5: Conducting a Cost Structure Analysis of the New Health Literacy Intervention Programs for Rural Communities Surrounding the Protected Area

#### Overview

Phase 5 is a continuation of Phase 4. We will conduct a cost analysis for every intervention program listed in Phase 4.

#### Data Collection Procedure

The cost structure will be prepared based on advice from experienced agencies or individuals in the field. A list of intervention activities will be presented as an alternative to the policy makers or any agencies who are interested in improving health literacy among the PNP community.

Economic data on providers' costs will be collected for the financial year 2021 to 2022. The data obtained will contain information on the costs incurred at the intervention and funding agency levels. When valuing economic costs, the opportunity cost of each resource will be considered, encompassing all resources used in the intervention, including those that are donated or subsidized. This approach ensures a uniform method of data collection, enabling reliable cost comparisons among the various interventions.

#### Data Analysis Procedure

The cost analysis will analyze expenses based on the cost unit, source data, and frequency, with comparisons made across the intervention programs. The cost unit pertains to the expenses associated with each intervention per patient per year. Simultaneously, the sources of data serve as evidence for the interventions, and frequency refers to the number of interventions required annually.

### Data Management and Analysis

#### Data Security

At the end of each day of data collection, the project manager will gather all interviewers and conduct a postmortem session. Any loopholes identified during the process will be addressed immediately to avoid further mistakes and invalidate the collected data. In addition, the project manager will verify if the number of answer booklets corresponds with the number of households surveyed. Upon verification, the answer booklets will be placed in a safe box, while the consent form will be kept in a separate secure box to avoid any information breach. Only the project manager and the principal investigator have access to these secured boxes. Similarly, all recorded videos and voices from FGD and interviews will be safely placed in a Dropbox (Evenflow Inc) for analysis.

#### Data Entry and Analysis

Data entry from the survey form will be completed within 2 to 4 weeks after completing the groundwork, depending on the number of respondents. Once all data have been entered into SPSS software, we will perform a random cross-checking of the data set to maintain data accuracy. The cross-checking will be set at 20% of the overall questionnaires entered [25]. Data analysis will comprise both descriptive and analytical analyses. Descriptive analysis will be performed on the population's sociodemographic characteristics, BMI measurements, expenditure, utilization of health care goods and services, modifiable lifestyle factors, and reported quality of life. The analytical examination will be conducted to determine the association between sociodemographic characteristics, health status, and socioeconomic status with health literacy status. The results will be analyzed using SPSS software. The data and results will be presented to and validated by field experts and stakeholders of PNP in the form of reports and seminar-sharing sessions.

### Ethical Considerations

Ethics approval for this study was granted by the Universiti Sains Islam Malaysia (USIM) Research Ethics Committee (USIM/JKEP/2022-216). Each respondent will sign a written informed consent form before the interview. Academic journals, conferences, and stakeholder seminars will be used to communicate the findings of this research. The protocol received approval from the International Standard Randomized Controlled Trial Number Registry (ISRCTN; 40626062).

## Results

The project was funded by USIMl and strategic research partnership grants. Enrollment for the overall project is ongoing. We are conducting a systematic review of health literacy in protected areas and conducting a cross-sectional study based on the survey results to determine the determinants of health literacy in protected areas. Findings from this study will be used to develop a sustainable health intervention module with an effective cost that may be adopted in all protected areas in Malaysia. The first results are expected to be submitted for publication in 2024.

## Discussion

### Anticipated Findings

The conservation policy of the protected areas has limited opportunities for local communities to harvest natural resources from the ecosystem. Their quality of life has been affected due to inferior education, health, and living conditions compared to communities living in urban areas. This disparity has resulted in challenges for the community in accessing health facilities and infrastructure, thereby limiting their health literacy level. Their remote location and distance from main roads have contributed to this low health literacy. To our knowledge, few studies have been conducted on health literacy and health issues among individuals in protected areas from 4 perspectives: government entities, knowledge institutions, civil agencies, and local communities. Accordingly, this paper proposes a study to investigate the health literacy and health status of communities within and near PNP. In accordance with the recommendations made by the PNP shareholders, this study aims to develop a health intervention program that is efficient and appropriate for community implementation. Health literacy research among people living in protected areas is essential for a deeper understanding of their needs for access to health care, illness prevention, health promotion, and health care. This understanding will guide the development of effective strategies to meet these requirements.

### Strengths and Limitations

This study’s strength lies in its mixed methods analysis, employing a triangulation process to converge and validate both qualitative and quantitative data [27]. Moreover, this study will be conducted in multiple phases to discuss the issue comprehensively. This structured approach facilitates incremental progress [28]. Phases 1 and 2 are conceptual phases where the issue is identified and validated through a systematic review by stakeholders in PNP, such as government agencies, knowledge institutions, and civil society organizations. Phase 3 will design and plan the questionnaire and examine the health literacy status and its influencing factors among the community. The stakeholders in the FGD in phase 4 will validate the results of phases 1 to 3. Using these results, the stakeholders will discuss and design an effective health literacy intervention program. Finally, phase 5 will involve the dissemination of the health literacy intervention program to improve the health literacy status in the community. Moreover, a cost analysis will be calculated to analyze the cost of the program.

The study’s limitations became apparent during the FGD session with the stakeholders, as the invited participants did not attend. Consequently, we missed capturing unique macro-level perspectives. Another limitation is that the stakeholders’ views may not be representative of all who live inside or near the protected area in Malaysia or globally. Additionally, the study did not address the distinctions between locals and outsiders. Access to rural areas is difficult logistically, and the potential presence of wildlife, such as tigers, elephants, and bears, further complicates the research process.

### Conclusions

In conclusion, this study will determine how different ecosystems, especially in protected areas, impact the health literacy of the local community using a mixed methods approach. The phases of the data collection process, incorporating both qualitative and quantitative methods, aim to comprehensively address the issue of health literacy in the local community. We will use these findings to disseminate a health intervention program, along with a cost structure, to improve health literacy among the protected area's community. The mixed methods study serves as a structured investigative model for evaluating and improving health literacy, health status, and well-being among all protected areas and communities worldwide.

## Appendix

Multimedia Appendix 1 Peer-review reports from the Universiti Sains Islam Malaysia (USIM).

## Abbreviations

FGD: focus group discussion
ISRCTN: International Standard Randomized Controlled Trial Number Registry
JAKOA: Jabatan Kemajuan Orang Asli
LFA: Logical Framework Analysis
NGO: nongovernmental agency
OR: odds ratio
PERHILITAN: Pahang Department of Wildlife and National Parks
PICO: Population, Intervention, Comparison, and Outcome
PNP: Pahang National Park
PRISMA: Preferred Reporting Items for Systematic Reviews and Meta-Analyses
USIM: Universiti Sains Islam Malaysia

## Data Availability

The data sets generated in this study are available from the corresponding author upon reasonable request.
