# Supplementary material for: The Impact of the Ecosystem on Health Literacy Among Rural Communities in Protected Areas: Protocol for a Mixed Methods Study
Source: JMIR Res Protoc. 2024 Jan 29;13:e51851. doi: 10.2196/51851 (PMC10862236; doi:10.2196/51851)
Supplement: Multimedia Appendix 1 [file resprot_v13i1e51851_app1.pdf]

(PPS/QT- CHAIRMAN)

PUSAT PENGAJIAN SISWAZAH<br>Center for Graduate Studies<br>USIM مركز الدراسات العليا	CHAIRMAN REPORT ON QUALIFYING TEST

QUALIFYING TEST DETAILS

**Date :**	26 January 2023	**Time :**	2.00 pm
**Semester :**	3		

CANDIDATE PARTICULAR

**Name :**	Nor Aziah Binti Abd Kadir
**Matric No. :**	4211371
**Program :**	Doctor of Philosophy (Medical Science)
**Title :**	The Impact of Ecosystem Diversity On Health Literacy Among Rural Communities Surrounding The Protected
**Supervisor(s) :**	1. DR. AMIRAH BINTI AZZERI
	2. DR. MOHD HAFIZ BIN JAAFAR
	3. PROF. MADYA DR. ZURINA BINTI KEFELI@ ZULKEFLI

QT COMMITTEE

**Chairman :**	Prof. Dr. Zairina Binti A. Rahman
**Examiner 1 :**	Prof. Madya Dr. Asral Wirda Ahmad Asnawi
**Examiner 2 :**	Dr. Tengku Madeehah Tg. Mohd
**Examiner 3 :**	

SECTION A : SUMMARY OF CHAIRMAN EVALUATION REPORT

PROPOSAL REPORT & PRESENTATION

Chapter 1: Introduction

Comments:

General: Ecosystem diversity is very broad terms that studies interaction of many types of ecosystem (human, animals, crops,aqua, air etc) and effect to human. This study should explain and focus on which part of ecosystem. Introduction and statement of problem focus much on poverty issues. There was lack of discussion on other related issues.
Linkbetween PA policy and health literacy is not clear.
Statement of problem: (page 9) Explain how illegal activities in NP contribute to bad impact to health status of locals.
(page 10): children in NP has poor education status and difficult to bring to school. Discuss the reasons
(page 12): Batek students separated from others with their own syllabus. Is it benefit more to the students? Improve their education?

What is current program to improve health literacy. How this study can benefit to current program

Operational definition: Definition that will be used for this study and not general definition
Eg: ecosystem biodiversity. Which type of ecosystem will be studied and what type of interaction
Health literacy. How this research categories health literacy and explain if any scoring involve.

Chapter 2: Literature Review

Comments:

Discuss Malaysian effort for health literacy among indigenous or underprivilege population. What is the progress with The National Health Literacy Policy.

Chapter 3: Methodology

Comments:

- Phase 2: what organization will be selected and who will be the target sample
- Phase 3:
- Study location: Pahang National Park. a) Explain justification to choose this location
- Who is the study population? PNP population (indigenous) , Malay community along the river
- Malay and indigenous, both has different sociodemographic, social life, economy. Will this factors affect outcome of study
- Inclusion and exclusion criteria. How to ensure respondents fulfil this criteria (eg; ID card)
- Indigenous are known to be very mobile and not staying in one place for long. How to overcome this problem
- Tools of study: Validation

Presentation

Comments:

Candidate presented clearly

SECTION B: QUALIFYING TEST RESULT

PART I: RESULT	
Please tick (/) one of the following boxes, as necessary.	
	Accepted and recommended to candidacy **(Please fill up Part III only)**
/	Conditional acceptance subject to minor corrections **(Please fill up Part II & III)**
	Conditional acceptance subject to major corrections **(Please fill up Part II & III)**
	Rejected and not recommended to proceed of which the candidate will be subjected to repeat the QT **(Please fill up Part IV only)**

PART II: DURATION FOR CORRECTION			
Please tick (/) the number of months that the corrected proposal must be submitted.			
/	3 months		6 months

PART III: PROPOSAL CORRECTION ENDORSEMENT			
Please tick (/) any of the following, as necessary.			
	Examiner 1		Examiner 2
	Examiner 3	/	Supervisor

PART IV: RE-QT			
Please tick (/) the number of months that the student is allowed to complete his/her correction.			
	6 months		12 months

DECLARATION<br><br>I hereby declare and certify that the report details of the student are as stated in this form.	
**Signature :**	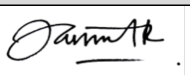
**Name :**	PROFESSOR DR ZAIRINA A RAHMAN<br>Faculty of Medicine and Health Sciences<br>Universiti Sains Islam Malaysia (USIM)<br>(MMC NO: 33642) (NSR NO: 137506)
**Position :**	**Chairman**
**Date :**	26 January 2023
